# The Combination of Clindamycin and Gentamicin Is Adequate for Pelvic Inflammatory Disease: A Retrospective Cohort Study

**DOI:** 10.3390/jcm10184145

**Published:** 2021-09-14

**Authors:** Li-Yeh Chen, Tomor Harnod, Yu-Hsun Chang, Hsuan Chen, Dah-Ching Ding

**Affiliations:** 1 Department of Obstetrics and Gynecology, Hualien Tzu Chi Hospital, Buddhist Tzu Chi Foundation, Tzu Chi University, Hualien 970, Taiwan; 103311147@gms.tcu.edu.tw (L.-Y.C.); amalfi33@hotmail.com (H.C.); 2 Department of Neurosurgery, Hualien Tzu Chi Hospital, Buddhist Tzu Chi Foundation, Tzu Chi University, Hualien 970, Taiwan; tomorha@yahoo.com.tw; 3 Department of Pediatrics, Hualien Tzu Chi Hospital, Buddhist Tzu Chi Foundation, Tzu Chi University, Hualien 970, Taiwan; cyh0515@gmail.com; 4 Institute of Medical Sciences, Tzu Chi University, Hualien 970, Taiwan

**Keywords:** clindamycin, gentamicin, metronidazole, pelvic inflammatory disease

## Abstract

Pelvic inflammatory disease (PID) affects 4.4% of women aged 18–44 in the United States, and may cause infertility if it is ineffectively treated. A combination of clindamycin and gentamicin is generally used for the treatment of PID. The benefit of adding metronidazole into the treatment combination still remains unclear, and this study was designed to evaluate its effectiveness. We retrospectively included 107 women who were diagnosed with PID from May 2013 to September 2020 in a single hospital. Based on their used antibiotic regimens, the patients were divided into three groups—those who were treated with clindamycin + gentamicin (group 1, *n* = 46), those who took regular antibiotics plus metronidazole (group 2, *n* = 27), and others (group 3, *n* = 34). Primary outcomes included the rates of taking surgery after failed antibiotics, occurrence/rupture of tubo-ovarian abscesses, and readmission within the following 6 months of first treatment. Secondary outcomes to assess were the length of stay (LOS) and expenditure for PID. There were no significant differences in the surgical rates, readmission rates, LOS and expenditure noted between the three groups. Subgroup analysis showed that visual analogue pain scores being 5 or more would increase the LOS by 3.83 days (*p* < 0.001), and body temperature > 38.3 °C or more would increase the treatment total expenditure (*p* < 0.001). Our study results suggest that the combination of clindamycin + gentamicin is a convincible treatment protocol for PID.

## 1. Introduction

Pelvic inflammatory disease (PID) refers to inflammation of the upper genital tract, involving the uterus, fallopian tubes, and/or ovaries [1]. PID usually arises from the pathogens such as *Chlamydia trachomatis*, *Neisseria gonorrhoeae*, and vaginal microorganisms (e.g., anaerobic organisms, enteric Gram-negative rods, streptococci, genital mycoplasmas, and *Gardnerella vaginalis*), those ascend from the lower genital tract and spread to the endometrium of the uterus, and into the fallopian tubes and ovaries [2]. The acute morbid effects of PID include abdominal pain, vaginal discharge, dyspareunia, and abnormal menstrual bleeding. However, chronic complications of PID include infertility, ectopic pregnancy, and chronic pelvic pain [3]. The prevalence of PID among sexually experienced women of reproductive age (18–44 years) in the United States was 4.4% in 2013–2014 [4]. The financial burden of PID highlights the importance of effective management of the disease. For each case of PID, an average treatment cost of USD 3202 is currently estimated [4].

The current guidelines of Centers for Disease Control and Prevention present several parental regimens for the inpatient treatment of PID, including cefoxitin + doxycycline, cefotetan + doxycycline, clindamycin + gentamicin or ampicillin/sulbactam + doxycycline. To prevent chronic sequelae of PID, treatment regimens recommended empiric, broad-spectrum coverage of potential pathogens, particularly in patients presenting severe disease with hospitalization. Although *Chlamydia trachomatis* and *Neisseria gonorrhea* have been frequently identified in patients with PID, the pathogens would be unknown among up to 70% of cases. Moreover, anaerobic bacteria have been isolated from the upper genital tracts in women with PID, and in vitro studies have revealed that some anaerobes (e.g., *Bacteroides fragilis*) can cause tubal and epithelial destruction [5,6]. Whether a routine add-on of metronidazole to the therapeutic regimen of PID is necessary has been discussed for years. The 2015 CDC guideline stated that the addition of metronidazole to the ceftriaxone (or cefoxitin) plus doxycycline is a considerable option [7]. The 2017 European guideline recommends adding metronidazole into the treatment regimen when antibiotics would be switched from intravenous or intramuscular routes to oral taking [8]. A randomized controlled trial of ceftriaxone and doxycycline for acute PID further suggested that metronidazole should be routinely given [9]. However, a systematic review from the Cochrane database did not show any clear evidence for the benefit of using metronidazole in ones with PID [2,10].

To clarify the benefits of routine use of metronidazole as one of the first-line treatments for PID, we compared the pre-discharge outcomes in inpatients who were treated with various combinations of antibiotics at our hospital.

## 2. Methods

### 2.1. Study Design

**Figure 1:**
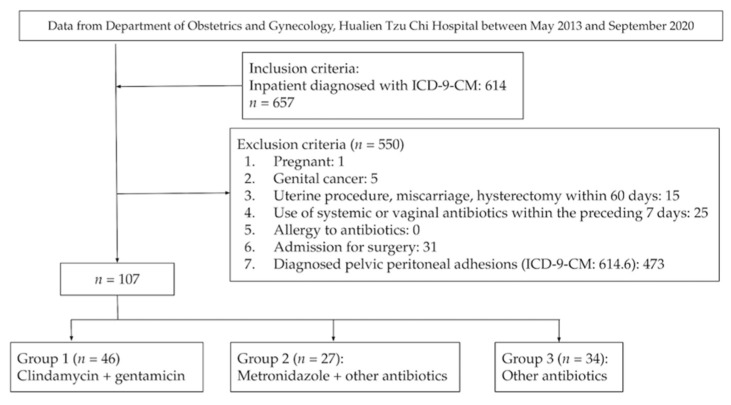
Flow chart of study design.

This was a retrospective study using data from Hualien Tzu Chi Hospital, Hualien, Taiwan. The diagnostic and treatment codes in the database were based on the International Classification of Disease, Ninth Revision, and the Clinical Modification (ICD-9 CM) during the study period. We reviewed inpatient records from May 2013 to September 2020. A total of 657 patients were noted with PID diagnosis (ICD-9-CM codes 614) during the period. Patients who were pregnant, had genital cancer, had undergone uterine procedure/miscarriage/hysterectomy within the past 60 days, had been administered systemic or vaginal antibiotics within the past 7 days, ever been admitted for surgery or diagnosed with pelvic peritoneal adhesions (ICD-9-CM codes 614.6) were excluded from the study. Therefore, 550 patients were excluded and the PID group finally consisted of 107 individuals (Figure 1).

The 107 individuals were divided into three groups based on their antibiotic regimens during hospitalization. Group 1 consisted of patients who were treated with a classic combination of clindamycin + gentamicin. Group 2 consisted of patients who were treated with metronidazole plus clindamycin + gentamicin or metronidazole plus other combinations. Patients in this group might be initially treated with different regimens, but received add-on metronidazole during the following admission days. For example, metronidazole was sometimes added to ceftriaxone + doxycycline, ciprofloxacin, and cephradine + gentamicin. Group 3 consisted of the rest of the patients who did not belong to group 1 or group 2.

The study protocol was approved by the Research Ethics Committee of Hualien Tzu Chi Hospital (IRB-109-212-B). All methods were performed in accordance with the relevant guidelines and regulations. Informed consent was waived due to low risk and approved by the above Committee.

### 2.2. Outcome Measurement

The primary outcomes of our study were the surgical rate after antibiotic treatments during hospitalization, the occurrence rate of the tubo-ovarian abscess (TOA), and the readmission rate in 6 months after we completed antibiotic treatments. The secondary outcomes were the length of stay (LOS) and the expenditure during hospitalization. Moreover, age, body mass index (BMI), smoking habits, history of pregnancy, use of intrauterine devices (IUD), number of days if pelvic pain noted with visual analogue scale (VAS) ≥ 5, number of days when body temperature (BT) being >38.3 °C, white blood cell (WBC) count and C-reactive protein (CRP) levels at admission, were included as covariates for further analysis. Underlying disease was selected as yes or no. The WBC counts were classified into two categories: >10,600/µL or ≤10,600/µL.

### 2.3. Statistical Analysis

All analyses were performed using the SPSS Software version 17.0 (SPSS, Inc., Chicago, IL, USA). Covariates and baseline morbidities in the three study groups were expressed as frequencies, proportions, or means ± standard deviations. Differences in continuous variables between groups were analyzed by one-way ANOVA and post hoc test with Bonferroni correction. Differences in categorical variables between the groups were analyzed by the chi-squared test. Logistic regression was used to analyze the association between primary outcomes (except TOA complications, due to its low incidence) and covariates. The odds ratios (ORs) and 95% confidence intervals (CI) were calculated. The associations between secondary outcomes and covariates were analyzed by linear regression, presented as β and 95% CIs. Differences were considered statistically significant when *p* < 0.05.

## 3. Results

This study examined 107 PID patients who were divided into three groups based on their antibiotic regimens during hospitalization. Table 1 shows the demographic characteristics of the three groups. The average age at diagnosis of PID of patients in group 2 was 43.96 ± 14.05 years, which was significantly higher than that of patients in group 1 (35.98 ± 11.61) (*p* = 0.03, post-hoc: 1 < 2). Compared with two other groups, the percentage of patients from group 2 who had previously been pregnant was also higher (96.3%, *p* = 0.03). The patients in group 3 reported a higher number of days with BT > 38.3 °C (0.58 ± 1.20) than those in group 1 (0.11 ± 0.38) (*p* = 0.03, post-hoc: 1 < 3). The surgical rates during hospitalization were 4.3%, 3.7%, and 8.8% (*p* = 0.66) for groups 1, 2, and 3, respectively. The rates of readmission within 6 months after discharge were 2.2%, 14.8% and 5.9% (*p* = 0.10) for groups 1, 2, and 3, respectively. The LOS were 4.78 ± 2.15, 6.11 ± 2.31, and 6.76 ± 7.16 months (*p* = 0.13) and the expenditures during hospitalization were New Taiwan Dollars (NTD) 14,857.87 ± 9095.32, 26,184.15 ± 23,164.93, and 33,078.88 ± 37,446.40 (*p* = 0.005, post-hoc: 1 < 3) for groups 1, 2, and 3, respectively (Table 1).

Logistic regression analyses of the factors associated with the risk of surgery during hospitalization and readmission after antibiotics treatment were performed. None of the covariates, except for age, have any significant influence on these two primary outcomes. A one-year increase in age increases the surgical rate during hospitalization by 1.19-fold. In this study, we disregarded TOA complications as a primary outcome because the number of patients involved was too small (Table 2).

Linear regression was analyzed for the factors associated with the risk of secondary outcomes (LOS and expenditure during hospitalization). Subgroup analysis showed that once VAS ≥ 5 would increase LOS by 3.83 days (95% Cl = 2.76, 4.89 *p* < 0.001), and BT > 38.3 °C would increase the hospitalization expenditure by NTD 18,276.24 (95% CI = 12,588.62, 23,963.85, *p* < 0.001). Moreover, a one-year increase in age was found to increase the LOS by 0.12 days (0.05, 0.19; *p* = 0.001) and hospitalization expenditures by NTD 458.91 (41.64, 876.18; *p* = 0.03) (Table 3).

No significant differences in the primary and secondary outcomes were noted among the three groups. TOA could not be compared among the three groups due to the small numbers (Table 2 and Table 3).

Table 4 shows antibiotic regimens in groups 2 and 3. There were seven patients receiving Clindamycin + Gentamicin + Metronidazole and 12 patients receiving Cefazolin + Gentamicin + Metronidazole in group 2. The antibiotics regimens were diverse in group 3.

Table 5 shows bacteria growth and patient who had culture rate in each group. The most common bacteria isolated were *Prevotella bivia* (22.22%), *Bacteroid fragilis* (25%), *Escherichia coli* (25.29%) in groups 1, 2, and 3, respectively. The patient who had culture rate was 17.39%, 33.33%, and 55.88% in the group 1, 2, and 3, respectively (Table 5).

## 4. Discussion

Early administration of appropriate antibiotics plays a significant role in preventing short- and long-term sequelae of PID. Metronidazole is a broad-spectrum antibiotic that exhibits activity against anaerobes, protozoa, and microaerophilic bacteria [11,12]. Anaerobes prevalent in the United States exhibit low resistance to metronidazole [13], and the resistance rates are generally low in most countries. The side effects of systemic metronidazole include gastrointestinal symptoms, most of which are not fatal. As mentioned earlier, there is still doubt about adding metronidazole to first-line treatment regimens for PID [9,10,14]. The CDC guidelines for PID treatment state that the addition of metronidazole to treatment regimens is an option [7]. On the contrary, the 2017 European PID treatment guidelines recommended its routine addition to oral treatment regimens [8].

This study implied convincing effectiveness of the combination of clindamycin + gentamicin in the treatment of PID. Further regimens containing metronidazole were not found to be superior in terms of the primary or secondary outcomes. Therefore, we suggest that the addition of metronidazole to anti-PID medications is not initially necessary; the combination of clindamycin + gentamicin should generally be effective in treating PID. Our results were consistent with the findings of an abridged version of a Cochrane systematic review and meta-analysis of randomized controlled trials evaluating PID treatment regimens [2,10]. In our study, we reviewed inpatients diagnosed with PID between May 2013 and September 2019. The majority of the patients visiting this hospital were Taiwanese, therefore, the results may only be applicable to patients of Asian ethnicity. However, the average age of patients in group 2 in this study was significantly higher than those in group 1. The percentage of patients in group 2 who had previously been pregnant was also greater, possibly as a result of the higher mean age.

The formation (one patient) or rupture (two patients) of TOA after admission was not common in this study. There is limited literature on the epidemiology of TOA following an episode of PID. Annually in the United States, approximately one-third of the PID cases have been found to concurrently have a TOA [15]. Furthermore, only approximately 15% of the patients with TOA presented with the signs and symptoms that suggest TOA rupture [16]. Due to the small number of TOA-related complications in this study, this factor could not be significantly analyzed.

Medical treatment with antibiotics is effective in 70% of patients [17]. The regimen of antibiotics also consists of broad-spectrum antibiotics including cefoxitin with doxycycline or clindamycin with gentamicin [18]. Once antibiotics are not effective in treating TOA, surgical management should proceed. Laparoscopy or laparotomy can be performed to drain the abscess and promote early recovery [17,19]. Ultrasound/computer tomography-guided drainage also can be performed for TOA treatment. The success rate of drainage is reported between 83% and 100% [20]. Taken together, TOA is a serious complication of PID. Broad-spectrum antibiotics can be given first. If antibiotics are not working, surgical or ultrasound-guided drainage should be performed.

*Prevotella bivia* is an anaerobic, Gram-negative, bile-sensitive rod belonging to the genus Bacteroides [21]. *Prevotella bivia* may harbor metronidazole-resistant characteristics which may be caused by *Nim* gene mutation [22]. *Bacteroid fragilis* and *E. coli* are also enteric pathogens and have an important role in acute PID [23]. In our study, the above-mentioned pathogens were the most common pathogens in each group, respectively.

Pelvic pain is generally evaluated by physicians with a VAS score based on patients’ facial expressions. A previous study reports the classification of PID patients with normal vital signs as mild or moderate PID when VAS is <5/10 or ≥5/10, respectively [24]. Therefore, we set a VAS pelvic pain score ≥ 5 as the cut-off value and VAS score ≥ 5 was reported for 27% of the participants in this study. Subgroup analysis revealed that a VAS score ≥ 5 resulted in a 3.83-day increase in the LOS. These findings indicate that the VAS pelvic pain score may be a potential prognostic factor in PID patients, during or after medical treatment courses.

This retrospective cohort study provides preliminary data supporting the combination use of clindamycin and gentamicin, without the addition of metronidazole for the treatment of PID inpatients. Further studies are required to account for other variables, such as drug side effects, including nausea, vomiting, and vaginal discharge. However, our study did have some limitations. Firstly, as this is a retrospective study, it carries information bias and selection bias. Physicians may have followed up with patients experiencing symptoms of PID more closely, leading to over-diagnosis. To minimize the impact of this factor, we intended to include the patients who were hospitalized only. Secondly, the overall conclusion of our study should be interpreted with care, since the study used a small sample size of only 107 patients. Thirdly, the VAS pelvic pain score may be influenced by the use of different forms of analgesics during hospitalization.

## 5. Conclusions

Early administration of appropriate antibiotics plays a significant role in preventing long-term sequelae of PID. This study showed that the combination of clindamycin + gentamicin is suitable for the treatment of PID. It is not necessary to add metronidazole to PID regimens routinely. The VAS score and body temperature of the patients were associated with their LOS and expenditure, respectively. The relationship between PID and different types of expenditure during hospitalization warrant further investigation in the future.

## Figures and Tables

**Table 1:** Basic characteristics of patients in the three groups (*n* = 107).

	Group 1	Group 2	Group 3	Total	*p*-Value	Post-Hoc
Number	46	27	34	107		
Age	35.98 ± 11.61	43.96 ± 14.05	42.15 ± 15.44	39.95 ± 13.87	0.03 *	1 < 2
BMI	25.25 ± 6.17	23.04 ± 4.43	25.05 ± 6.18	24.63 ± 5.81	0.26	
Smoker (%)	14 (30.4%)	7 (25.9%)	8 (23.5%)	29 (27.1%)	0.78	
Ever pregnant (%)	32 (69.6%)	26 (96.3%)	26 (76.5%)	84 (78.5%)	0.03 *	
Currently had IUD (%)	9 (19.6%)	3 (11.1%)	2 (5.9%)	14 (13.1%)	0.20	
Underlying disease (%)	14 (30.4%)	12 (44.4%)	11 (32.4%)	37 (34.6%)	0.45	
Days with VAS of pelvic pain ≥ 5	0.36 ± 0.71	0.26 ± 0.45	0.61 ± 1.12	0.41 ± 0.82	0.22	
Days of BT > 38.3 °C	0.11 ± 0.38	0.30 ± 0.54	0.58 ± 1.20	0.30 ± 0.79	0.03 *	1 < 3
WBC	12,842.44 ± 4121.82	13,944.44 ± 4897.31	13,336.76 ± 4649.52	13,281.70 ± 4477.62	0.60	
WBC > 10,600 (%)	35 (77.8%)	19 (70.4%)	22 (64.7%)	76 (71.7%)	0.44	
CRP	7.13 ± 7.76	11.03 ± 8.76	8.94 ± 7.68	8.71 ± 8.07	0.17	
Complications of TOA (%)	1 (2.2%)	0 (0.0%)	1 (3.0%)	2 (1.9%)	1.00	
Surgery during hospitalization (%)	2 (4.3%)	1 (3.7%)	3 (8.8%)	6 (5.6%)	0.66	
Readmission within 6 months (%)	1 (2.2%)	4 (14.8%)	2 (5.9%)	7 (6.5%)	0.10	
Length of stay	4.78 ± 2.15	6.11 ± 2.31	6.76 ± 7.16	5.75 ± 4.47	0.13	
Expenditures	14,857.87 ± 9095.32	26,184.15 ± 23,164.93	33,078.88 ± 37,446.40	23,505.76 ± 25,825.62	0.005 *	1 < 3

IUD: intrauterine device, BMI: body mass index, VAS: visual analogue score, BT: body temperature, TOA: tubo-ovarian abscess, WBC: white blood cell, CRP: C-reactive protein. * *p* < 0.05.

**Table 2:** Factors associated with primary outcomes (*n* = 107).

	Surgical Rate during Hospitalization		Readmission within 6 Months	
	Odds Ratio (95% CI)	*p*-Value	Odds Ratio (95% CI)	*p*-Value
Age	1.19 (1.02, 1.40)	0.03 *	0.94 (0.83, 1.07)	0.38
Group	-	-	-	-
Group 1	1.00 (Reference)	NA	1.00 (Reference)	NA
Group 2	0.15 (0.00, 11.85)	0.40	6.54 (0.30, 142.96)	0.23
Group 3	0.24 (0.01, 7.50)	0.42	2.60 (0.09, 73.20)	0.57
Smoker (Yes vs. No)	39.15 (0.36, 4227.59)	0.13	0 (NA)	1.00
Ever pregnant (Yes vs. No)	0.24 (0.00, 15.11)	0.50	1.1 (0.04, 32.47)	0.96
IUD (Yes vs. No)	0 (NA)	1.00	0 (NA)	1.00
BMI	1.12 (0.86, 1.45)	0.41	1.07 (0.85, 1.34)	0.57
Has underlying disease (%)	0.93 (0.12, 7.17)	0.94	3.83 (0.45, 32.28)	0.22
Days of VAS of pelvic pain ≥ 5	2.96 (0.74, 11.76)	0.12	4.50 (0.63, 32.16)	0.13
Days of BT > 38.3 °C	4.50 (0.82, 24.71)	0.08	0 (NA)	1.00
WBC (>10,600 vs. ≤10,600)	0.23 (0.01, 5.99)	0.37	8.84 (0.32, 247.14)	0.20
CRP	0.97 (0.84, 1.11)	0.64	0.77 (0.54, 1.09)	0.14

IUD: intrauterine device, BMI: body mass index, VAS: visual analogue score, BT: body temperature, WBC: white blood cell, CRP: C-reactive protein. Statistical analysis: Logistic regression. * *p* < 0.05.

**Table 3:** Factors associated with secondary outcomes (*n* = 107).

	LOS		Expenditure	
	β (95% CI)	*p*-Value	β (95% CI)	*p*-Value
Intercept	0.82 (−4.12, 5.77)	0.74	−9560.63 (−38,256.47, 19,135.22)	0.51
Age	0.12 (0.05, 0.19)	0.001 *	458.91 (41.64, 876.18)	0.03 *
Group	-	-	-	-
Group1	1.00 (Reference)	NA	1.00 (Reference)	NA
Group2	0.04 (−2.02, 2.10)	0.98	4512.37 (−7440.75, 16,465.49)	0.46
Group3	−0.25 (−2.18, 1.68)	0.80	4979.54 (−6206.78, 16,165.86)	0.38
Smoker (Yes vs. No)	−1.07 (−3.06, 0.92)	0.29	4622.40 (−6917.31, 16,162.11)	0.43
Ever pregnant (Yes vs. No)	−0.77 (−2.80, 1.25)	0.45	−3765.01 (−15,522.36, 7992.34)	0.53
IUD (Yes vs. No)	−0.76 (−3.05, 1.53)	0.51	−6210.64 (−19,520.41, 7099.13)	0.36
BMI	−0.10 (−0.24, 0.04)	0.15	195.23 (−600.64, 991.11)	0.63
Underlying disease (Yes vs. No)	−0.35 (−1.62, 0.92)	0.58	562.16 (−6804.65, 7928.98)	0.88
Days of VAS of pelvic pain ≥ 5	3.83 (2.76, 4.89)	<0.001 *	4823.38 (−1339.23, 10,985.99)	0.12
Days of BT > 38.3 °C	0.55 (−0.43, 1.53)	0.27	18,276.24 (12,588.62, 23,963.85)	<0.001 *
WBC (>10,600 vs. ≤10,600)	−0.45 (−2.26, 1.36)	0.62	−7447.74 (−17,933.58, 3038.09)	0.16
CRP	−0.01 (−0.11, 0.10)	0.91	289.68 (−321.27, 900.62)	0.35

LOS: length of stay, IUD: intrauterine device, BMI: body mass index, VAS: visual analogue score, BT: body temperature, WBC: white blood cell, CRP: C-reactive protein. Statistical analysis: Linear regression. * *p* < 0.05.

**Table 4:** Antibiotic regimens in groups 2 and 3.

	Group 2 (*n* = 27)	Group 3 (*n* = 34)
Antibiotic regimens	Clindamycin + Gentamicin + Metronidazole (*n* = 7)	Clindamycin + Gentamicin + Doxycycline (*n* = 2)
	Ciprofloxacin + Metronidazole (*n* = 1)	Cefazolin (*n* = 4)
	Cefazolin + Gentamicin + Metronidazole (*n* = 12)	Imipenem (*n* = 2)
	Cefmetazole + Doxycycline + Metronidazole (*n* = 1)	Ceftazidime (*n* = 1)
	Ceftriaxone + Metronidazole (*n* = 2)	Cefmetazole (*n* = 1)
	Ceftriaxone + Doxycycline + Metronidazole (*n* = 3)	Imipenem + cefmetazole (*n* = 1)
	Flomoxef+ Metronidazole (*n* = 1)	Ertapenem (*n* = 3)
		Levofloxacin + (Sulfamethoxazole + Trimethoprim) (*n* = 2)
		(Piperacillin + Tazobactam) + Doxycycline (*n* = 1)
		Levofloxacin (*n* = 1)
		Cefazolin + Gentamicin (*n* = 1)
		Clindamycin + Doxycycline (*n* = 1)
		Ampicillin + Levofloxacin (*n* = 2)
		Flomoxef (*n* = 4)
		Ampicillin + Levofloxacin (*n* = 1)
		Amoxicillin + clavulanic acid (*n* = 1)
		Meropenem (*n* = 1)
		Levofloxacin+ Doxycycline (*n* = 1)
		Cefepime (*n* = 1)
		Ampicillin + Doxycycline (*n* = 2)
		Moxifloxacin (*n* = 1)
		Oxacillin + Clindamycin (*n* = 1)

**Table 5:** Bacteria growth and antibiotics resistance rate among each group.

	Group 1	Group 2	Group 3
Most common bacteria	*Prevotella bivia* (22.22%), *Bacteroid fragilis* (22.22%)	*Bacteroid fragilis* (25%)	*Escherichia coli* (35.29%)
Other bacteria	*Escherichia coli* (11.11%), *Peptostreptococcus species* (11.11%), *Fusobacterium nucleatum,* (11.11%), *Fusobacterium necorphorum* (11.11%), *Cutibacterium acnes* (11.11%)	*Escherichia coli* (12.5%), *Enterobacter aerogenes* (12.5%), *Enterococcus faecalis* (12.5%), *Gardnerella vaginalis* (12.5%), *Peptostreptococcus anerobius* (12.5%), *Prevotella bivia* (12.5%)	*Enterococcus* (11.76%), *Prevotella bivia* (11.76%), *Streptococcus* (11.76%), *Bacteroid fragilis* (5.88%), *Klebsiella pneumoniae* (5.88%), *Peptostreptococcus species* (5.88%), *Proteus mirabilis* (5.88%), *Streptococcus sanguinis* (5.88%)
Patients who had culture (%)	17.39%	33.33%	55.88%

## Data Availability

All relevant data were shown in the manuscript.
